# Design of a Freely Accessible Web Application (Instrument for the Measurement of Balance in Primary Education, IMEP) for the Assessment of Static and Dynamic Balance in Children Aged 6–9 Years Based on Force Platforms

**DOI:** 10.3390/jfmk9040281

**Published:** 2024-12-20

**Authors:** Julio Martín-Ruiz, Ignacio Tamarit-Grancha, Carlos Cordente-Martínez, Raúl Santamaría-Fernández, Concepción Ros Ros, Laura Ruiz-Sanchis

**Affiliations:** 1 Department of Health and Functional Assessment, Catholic University of Valencia, 46900 Valencia, Spain; julio.martin@ucv.es; 2 Department of Physical Preparation and Conditioning, Catholic University of Valencia, 46900 Valencia, Spain; ignacio.tamarit@ucv.es; 3 Department of Sports, Polytechnic University of Madrid, 28040 Madrid, Spain; carlos.cordente@upm.es; 4 Faculty of Sciences of Physical Activity and Sports, Catholic University of Valencia, 46900 Valencia, Spain; rasanfer@mail.ucv.es; 5 Department of Sports Management and Physical Activity, Catholic University of Valencia, 46900 Valencia, Spain; concepcion.ros@ucv.es

**Keywords:** motion analysis, physical activity, educational application, balance, primary education

## Abstract

**Background:** The proper development of balance is essential in the acquisition of a correct physical condition, as well as in the evolutionary follow-up at early ages, and its periodic evaluation is very relevant in the educational environment. **Objectives**: The objective of this research was to design an accessible web application for static and dynamic balance assessment, based on a force platform and motion analysis software. **Methods**: The Single leg balance test (SLB), Tandem balance test (TBT), and Y balance test (YBT) were performed on a sample of 75 children aged 6 to 9 years. **Results**: The results show that static balance is more complex at an older age, greater standing height, and with eyes closed (*p* < 0.001). Regarding the center of pressure (COP), its variability was greater in girls owing to a lower Total Force (TF) at the time of the test (*p* < 0.05). Parallel observation with the Kinovea software has made it possible to elaborate a scale from 1 to 10 points for integration into an open-access web application (IMEP) to assess static and dynamic balance. **Conclusions:** The creation of an ad hoc application for primary school teachers and students has been possible by using validated devices obtaining a rating scale, which facilitate the monitoring of students’ functional evolution and offers the possibility of scheduling physical education sessions with a preventive approach as well as a focus on improving physical condition.

## 1. Introduction

Balance is fundamental to the development of motor skills, body schemas, and tonic functions [1]. It is the basis of coordination and spatial–temporal control, always represented by an initial imbalance until the end of rebalance, particularly in sports movements [2,3,4]. The interaction of its variables, the base of sustenance, center of mass (COM) [5], and changing the center of pressure (COP) at each joint [6] is basic for an efficient and precise motor sequence. A balanced action [7] is defined by cooperation between the central nervous system as a regulator and the musculoskeletal system as the executor.

Balance control is primordial at early ages; up to three years of age, it allows the first tasks of displacement, position maintenance, and dynamic balance [8]. Between 4 and 5 years of age, there is no established relationship between static and dynamic balance [9] (ability to stand upright with or without movement, respectively) because of the maturation of brain areas related to motor control. This relationship is slightly better in girls, without being significant [10,11]. At eight and nine years of age, some studies relate children’s development to improved self-concept and self-efficacy [12] when the movement is clearer and more oriented, owing to the plasticity of the nervous system that leads to a great gestural improvement, highlighting the oculo-segmental, a qualitative change in the activities of balance and general coordination [13], which makes this age group the most suitable for static balance work [14]. From the age of 9 years, anatomical growth results in a motor imbalance that improves with adequate and continuous physical exercise [15] and reaches its peak at approximately 23 years of age [16].

The role of Physical Education teachers in consolidating this achievement is fundamental from the age of six years, guaranteeing greater motor richness with the application of different skills: physical–motor, socio-motor, and perceptual–motor [17]. Its systematic and structured work [18] provides benefits when prolonged for more than one month [19]. The use of interactive balance tools such as the MABC-2 Battery can aid in the early detection of coordination disorders [20]; thus, the integration of these types of resources in the classroom with an adequate evaluation and interpretation of data [16] is of added value and great support to the teaching staff.

Traditionally, balance assessment consists of measuring the time it takes to maintain a position, minimizing visual stimuli, as it depends on the functions of the vestibular and proprioceptive (somatosensory) systems [21,22,23]. Measurement techniques are complex and, being proprioceptive in nature, take the physiological mechanisms, influencing factors, and location of the variable within the system as a reference. The complex interactions between these elements make it difficult to analyze and measure their specific functions and characteristics [24]. Most current techniques assess the integrity and function of proprioceptive components with measurements along afferent and efferent pathways, the result of musculoskeletal activation, or a combination of the two [25].

The static tests include unipodal tests, such as the flamingo test [26], with its variant with eyes closed [27], and the bipodal ones, such as the tandem or Romberg’s test, which includes hands at the side and eyes closed [24]. In unipodal dynamics, the equilibrium test in T [28], the well-known Y balance test (YBT), and the Star Excursion Balance Test (SEBT) [29,30] are widely used in recovery protocols, with work suggesting the integration of other movement models [31]. In the case of bipodal tests, the Gesell balance test can be used [16], or tandem walking, which is also used in special populations [32]. In general, the differences between tests usually boil down to the placement of body segments and the execution time [33,34].

For accurate assessment, isolating dependent variables with direct measurements from medical applications, such as the Biodex Balance System [35], force platforms [36], pressure analysis, or the use of the baropodometer [5], is ideal for measuring symmetry, moment of force, COM and COP projection, plantar support, barycenter, or stabilometry in a static position [37]. However, in the educational field, this type of instrumentation is inaccessible owing to the cost and student ratio. Alternative tools, mainly mobile applications, have been designed to calculate the balance with increasing accuracy [38,39], taking advantage of the fact that, by default, these devices include an accelerometer but offer only one form of balance, are not open source, and are not globally accessible. A shortage of open and collective resources that measure the precision of validated instruments and can be integrated into the specific contents of the curriculum has been detected [40].

For these reasons, the objective of this research was to develop an objective and accessible tool that allows teachers and students between six and nine years of age to assess static and dynamic balance. This would allow obtaining an accurate measurement of different types of balance with different applications: school for teachers and students and personal monitoring adjusted to individual characteristics, with the incentive of being open and having a comparative reference with other cases of the same educational cycle.

## 2. Materials and Methods

### 2.1. Experimental Approach to the Problem

To develop an objective and accessible tool that allows Physical Education professionals to evaluate balance in children aged 6–9 years, a cross-sectional study was carried out using quantitative biomechanical tests and field evaluations with movement analysis instruments.

Five primary schools participated in this study and provided authorization. Informed consent was obtained from the parents/legal guardians of all participants. The timetable for measuring the tests agreed with the Physical Education teachers of each center. The study was designed in accordance with the Declaration of Helsinki to guarantee the fundamental rights of research on human subjects and was approved by the Ethics Committee of the Catholic University of Valencia (reference number: UCV/2015-2016/049).

### 2.2. Participants

The sample was selected based on the accessibility of the participating schools and was therefore non-probabilistic by convenience. The inclusion criteria were as follows: being a student from the 1st to 3rd year of Primary Education (6 to 9 years old), not suffering from any lower limb injury, and voluntary participation of the Physical Education teacher in the research. The reasons for exclusion were limited to non-compliance with the age of the study. The descriptive data for the sample are shown in Table 1.

### 2.3. Procedure

Informed consent was obtained (guardians/parents), and anthropometric measurements of weight (Seca 750, Hamburg, Germany) and height (Seca 213, Hamburg, Germany) were measured by a qualified professional (ISAK-I).

Leg length: With the knee extended, the talus (ankle) and greater trochanter of the femur (hip) were located, and the distance between the two was measured with a metallic tape measure. Next, the laterality of the lower extremities was identified using an accurate chopstick test [41].

Finally, self-adhesive markers were placed on the following bony landmarks: (1) acromion: starting from the distal third of the clavicle, the thumb was slid to its end point, and the marker was placed on the most prominent edge on the glenoid surface of the humerus; (2) navel: in the anterior frontal plane, the marker was placed in the hollow located in the abdomen; and (3) the external border of the greater trochanter of the femur, starting from the sagittal plane, and the most prominent lateral bony border was located. The thumb was placed, and the subject was asked to perform hip flexion to detect the pivot point [42] and (4) L3 lumbar vertebra: from the posterior frontal plane, in a standing position, both hands were placed on the edges of the pelvis located at the L4 lumbar vertebra by palpation, and one vertebra ascended and was located at L3.

**Figure 1:**
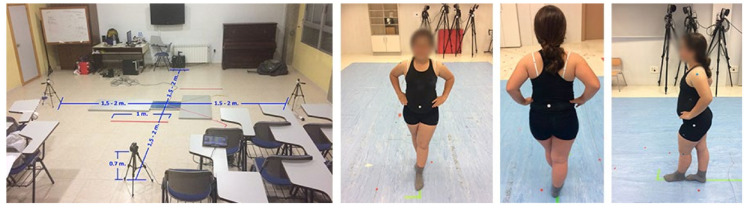
Adequacy of the measurement environment and joint markers. Note: (**Left**) Study environment; (**Center-left**): frontal marker, umbilicus; (**Center-right**): dorsal marker, lumbar L3; (**Right**): lateral markers, acromion, and greater trochanter.

To proceed with the measurement, the space was adapted in such a way that the portable force platform (Kistler, Model 9260AA, Winterthur, Switzerland) was placed in the center and with four cameras (GoPro Hero, Model 3+ Silver, San Mateo, CA, USA): one front (displacement of the umbilicus), one rear (of L3), and two lateral (variation of the greater trochanter and acromion) located at a distance of 2 m from the central point of the platform (150 × 250 mm) and at a height of 0.7 m. Finally, a camera perpendicular to the subject (Panasonic, Model HC-V130, Osaka, Japan) in HD (17 Mbps/VBR) with 1920 × 1080 px resolution for subsequent analysis using the Kinovea software (Kinovea, v2023.1, Bordeaux, France) and two 1m red marks visible from all cameras as a reference for this software (Figure 1).

A single measurement session was performed without prior standardized warm-up to avoid conditioning the tone of muscle activation during the test. They were shown the procedure of each test and were familiarized with each test for five minutes before performing the actual measurement and were able to ask the questions they needed. A total of 3 tests were performed with a total of 14 different balance positions: 4 unipodal support (single-leg balance, SLB) of 16 s with eyes open (2) and closed (2); 4 tandem balance test (tandem balance test, TBT) of 16 s, with eyes open (2) and closed (2); and 3 dynamic balance of 5 s (Y balance test, YBT), in the frontal (2), posteromedial (2), and posterolateral (2) directions [43]. In the cases of the SLB and TBT, they were always started with the right leg.

The objective of the SLB is to maintain balance with one leg on the reference marks placed on the platform [34], with the subject immobile with hands on the waist, repeating the test if there is support from both feet.

In TBT, the load differences in the lower extremities in the standing position were evaluated [44]. It is performed by touching the toe of the rear foot to the heel of the front foot and the hands on the waist. If a change in foot landing occurred, the test was repeated.

Finally, the YBT modified the SEBT, which is a valid and reliable tool for assessing unipodal dynamic balance [45]. Movements were performed in three different directions (frontal, posteromedial, and posterolateral), maintaining the position for 1 s without losing support, looking for symmetry on both sides of the body through the distances reached using the following formula [46]:


YBT = distance in frontal direction + distance in posteromedial direction + distance in posterolateral direction/(leg length × 3) × 10

**Figure 2:**
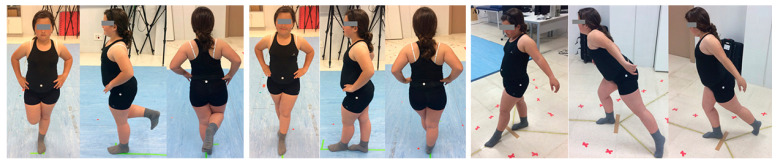
Representation of the balance tests used. Note: (**Left**): Single-leg test; (**Center**): tandem balance test; (**Right**): Y balance test.

The execution of each test is illustrated in Figure 2.

Data from the force platform were collected using the Bioware software (Kistler, v5.3.0.7, Winterthur, Switzerland) and exported as a .txt extension file, and signal processing was performed using MATLAB (2024b) (Mathworks Inc., Natick, MA, USA). The signal was digitally cleaned with a 10–50HZ bandpass filter to collect only the useful signals. The Root Mean Square (RMS) was then performed, and the data were segmented as follows: from Test 1 to Test 8 (SLB and TBT test), the first 3 and last 3 s of the 16 total were removed to avoid peaks at the start setting and fatigue at the end of the test. From records 9 to 14 (YBT), the initial 4 s corresponding to the performance of each movement was used. In all cases, the force and COP data along the X-, Y-, and Z-axes were collected.

**Figure 3:**
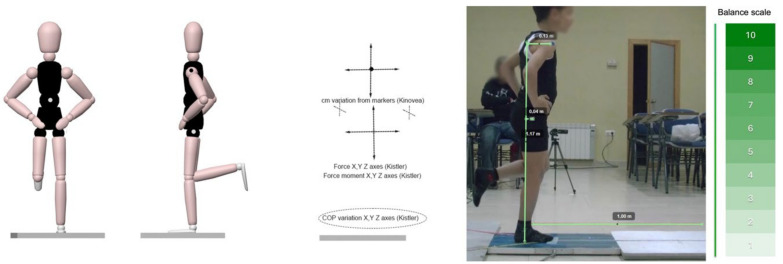
Study workflow. Note: (**Left**): Performance of the balance tests; (**Center-left**): registration of variables; (**Center-right**): analysis with the Kinovea software; (**Right**): conversion to the equilibrium rating scale.

Similarly, with the Kinovea software (no possibility of synchronization with the platform), the variations in the movement of each of the tests were collected with the cameras in centimeters (1920 × 1080 px minimum resolution). Each video was labeled to distinguish the number of attempts (from 1 to 14); whether it was a frontal or lateral camera, considering the joint markers, the cm of deviation in each case were noted to relate them statistically and establish a correct rating scale in the design of the web application designed ad hoc, as shown in Figure 3.

### 2.4. Web Application Design

A responsive web application (with functionality on mobile devices) was designed for use by teachers and students of Physical Education in primary education. The name ascribed to it was the Instrument for the Measurement of Balance in Primary Education (IMEP). It is hosted in the subdomain imep.giepafs.net/IMEP/, dependent on the main domain giepafs.net and its access is free.

The programming was performed on a Linux server, CentOS v.7.9.2009 (Core), in the PHP language (PHP, v8.1.29., Greenland), and MariaDB (MariaDb, v. 10.5.26., Tampere, Finland).

**Figure 4:**
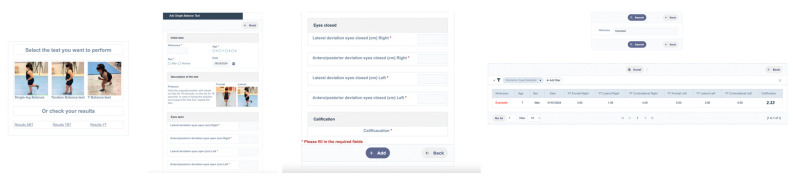
Sequence of use of the IMEP web application. Note: (**Left**): Application input interface; (**Center**): example of SLB test (two images); (**Right**): individual results search engine; * = mandatory answer.

It starts with a panel in which one can choose the balancing test to be performed. Once selected, there is an area in which to enter the user data; a nickname or pseudonym is preferred for data protection. In each field, the cm of deviation of the test is indicated, and at the end, a score of between 1 and 10 points is obtained, based on the research carried out on the forces platform (the value 0 was avoided as it is carried out for educational contexts). If each participant has several records and returns to the home page, you can see all the results by choosing the nickname, at which time the attempts and results of all the tests will be displayed (Figure 4).

### 2.5. Statistical Analysis

#### 2.5.1. Descriptive Analysis

The data were described as means and standard deviations, as well as medians and interquartile ranges for continuous quantitative variables. For qualitative variables, proportions and 95% confidence intervals were determined.

#### 2.5.2. Logistic Regression Models

To determine the causes of test complexity, YBT score, and changes in balance and movement, mixed-effects regression models were performed with the packages lme4 [47] v.1.1-30 and lmerTest [48] v.3.1-3, according to the protocol of Zuur and Ieno [49]. First, the error structure of the data was determined by fitting the beyond optimal model, which included the factors of interest in each case, along with test-related factors (test type, eyes open or closed, and dominance in the supporting foot), age, sex, and foot number.

Second, the random structure of the model was fitted using the participant ID, and coefficients were estimated using the REML method. Finally, a simplified optimal model was reported by estimating the coefficients using the REML method. To interpret the model, the coefficients and the results of the ANOVA were calculated using the car package [50] v.3.1.0. For significant categorical factors, post hoc comparisons were performed using the emmeans package [51] v.1.7.5.

All nested models were compared using the corrected Akaike information criterion (AICc) calculated using the MuMIn package [52] v.1.46.0. In all cases, the assumptions of the linear models were checked by visually inspecting the residuals and performing the Shapiro–Wilk (normality) and Levene (homoscedasticity) tests, as well as with the v.0.10.3 package [53]. The models were fitted with the package lme4 [47] v.1.1-30.

#### 2.5.3. Calculation of Scales

To obtain a score on a scale of 1 to 10 for each test (SLB, TBT, and YTest), the following procedure was followed:

### 2.6. Notation

In the following mathematical demonstration, a test is defined as a set of static or dynamic balance exercises. Thus, in the present study, there were three tests: SLB, TBT, and YTest, each of which was composed of four exercises.

#### 2.6.1. Reference Point Motion Calculation

In each exercise of a given test, displacement was measured at three reference points: the acromion, umbilicus, and trochanter. The Pythagorean theorem was used on the X-and Y-axes. Subsequently, three displacements were added to obtain the movement of a given exercise.

$$mvtu\;mbilicus.=\sqrt{{despl.X}^{2}umb.+{despl.Y}^{2}}umb.$$

In the case of the YBT, the score is calculated in the usual way:

YBT = distance in frontal direction + distance in posteromedial direction + distance in posterolateral direction/(leg length) × 10

#### 2.6.2. Total Movement and Gross Score Calculation

The movements of each exercise were summed to obtain the total movement of the test, which was used as a reference for the raw score of the test. Because a higher amount of movement indicates a lower performance, the value of the total movement is inverted. To conduct this, the total movement is subtracted from a constant (*cte*), thus ensuring that the resulting values are positive. The value of *cte* is determined for each test by attending to the range of variation in the amount of total motion.

$$Raw\;score=\left(mvt\;umb\;a+mvt\;umb\;b+mvt\;umb\;c+mvt\;umb\;d\right)+cte$$

In the case of the YBT, the scores are added together to obtain the raw score. In this case, a greater movement implies a better performance; so, there is no need to reverse the value. Since this score has a logarithmic scale, it is transformed, and a constant is subtracted, cte:

$$Raw\;score=log\left(YBT\;a+YBT\;b+YBT\;c+YBT\;d+YBT\;e+YBT\;f-cte\right)$$

#### 2.6.3. Adjustment of the Raw Score by Demographic Variables

To adjust the raw scores for demographic variables (sex, foot size, and age), a linear regression model was fitted to generate a correction factor for each participant. This was applied to the raw scores, as follows:

Adjusted score=Raw score−0.5·FC

where FC is the correction factor obtained from the linear regression model. It is multiplied by 0.5 to smooth its impact on the adjusted score.

#### 2.6.4. Rescaling to the Scale of 1 to 10

The adjusted score was rescaled to a range from 1 to 10 to obtain the final test score. This rescaling varied according to the test type.

$$Adjusted\;score=\frac{Adjusted\;score-min(Adjusted\;score)}{\max\left(Adjusted\;score\right)-min(Adjusted\;score)}\cdot 9-1$$

In all analyses, the following was used: *α* = 0.05.

The analyses were performed using R [54] v.4.2.2. Data tables were read with the openxlsx package [55] v.4.2.5 (for xlsx files) and/or haven [56] v. (for sav files). The graphics were constructed with ggplot2 [57] v.3.5.1 ggpubr [58] v.0.4.0, and other functions integrated in the mentioned packages.

## 3. Results

### 3.1. Analysis of the Complexity of Each Test

An equation was designed to determine the complexity of the exercise based on the beyond optimal model.


Complexity ~ FootSupport + Age + TestType × (Foot_Size + Sex) + EyesOpen × Foot_Size (1 | ID)

After fitting it to a gamma distribution with logarithmic linkage, the model presents a good fit of the residuals, complying with linearity, normality, homoscedasticity, and absence of outliers. Table 2 shows the six significant effects after the application of a type III ANOVA.

**Figure 5:**
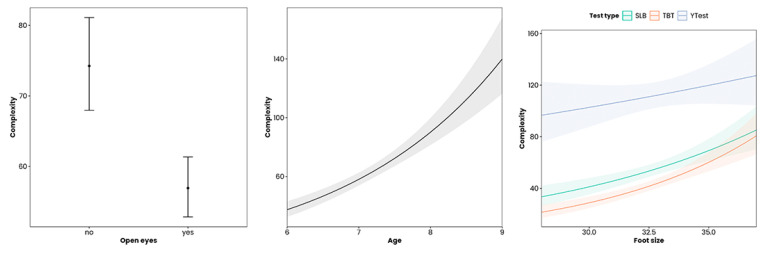
Variation in the complexity score as a function of eye opening, age, and standing size. Note: SLB = Single-leg balance. TBT = Tandem balance test.

As it can be seen, a greater age, foot size, and performing the test with eyes closed (in SLB and TBT) significantly conditioned the complexity, increasing the score of this variable by 1.3 ± 0.05 (Figure 5).

### 3.2. Effect of Leg Length on YBT

In the case of YBT, we used the beyond optimal model to calculate whether the leg length influenced the results using the following equation:


Complexity ~ FootSupport + Age + TestType × (Foot_Size + Sex) + EyesOpen ∗ Foot_Size (1 | ID)

Table 3 shows with a type II ANOVA that the scores are higher for anterior movement, lower for posterolateral movement, and intermediate for posteromedial movement.

### 3.3. Relationship Between Equilibrium and COP Modification

In addition, by applying the beyond optimal model, it was determined that the best structure for equilibrium is:


Balance ~ TestType + FootSupport + Eyes Open + FT + MT + COP_A + Foot_Size + Sex + FT + MT + COP_A + Sex + FT + MT + COP_A + Sex + Foot_Size + Sex + Sex +FT + MT + Sex + COP_A + Sex

Age + TestType: SizeFoot + TestType: Sex + EyesOpen:Size_Foot + EyesOpen:Sex + FT:Sex + MT + COP_A:Sex

Age affected balance, with older participants showing a better balance than younger participants (*p* < 0.001). The type of test affected the balance according to the foot size. The greater the number, the greater the static balance with eyes open (SLB and TBT) (*p* < 0.01) as well as sex, showing better results in girls in the case of SLB and TLB and neutral in YBT (Table 4).

**Figure 6:**
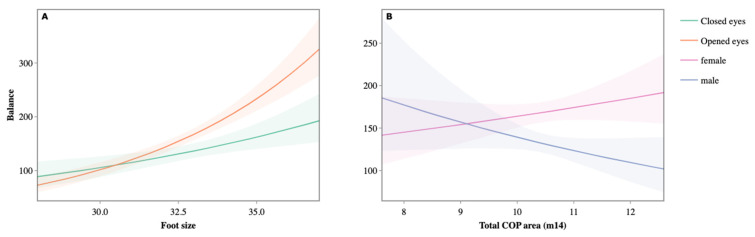
Variation in the balance score with eyes open or closed (**A**) and POP by sex (**B**).

In the case of the center of pressure (COP), the greater the variation, the higher the girls’ score in equilibrium, whereas the opposite phenomenon occurred in the case of boys, who improved their score when the COP was reduced (Figure 6).

### 3.4. Relationship Between Complexity and POP Modification

As in the previous case, through the beyond optimal model, it was determined that the best structure to establish the complexity was:


Complexity ~ TypeTest + FootSupport + Eyes open +FT + MT + COP_A + Foot_Size + Sex + Age + TestType: FootSize + TestType:Sex + FT:Foot_Size + MT:Sex + CO:A:Sex

Age was a determinant in assessing complexity, increased the older they get (*p* < 0.001), TBT, and foot size (*p* < 0.001), especially in girls (Table 5).

The application of force is fundamental for describing the complexity related to the foot size. It decreased with the increase in the force in each axis and with the increase in the foot size (Table 6).

**Figure 7:**
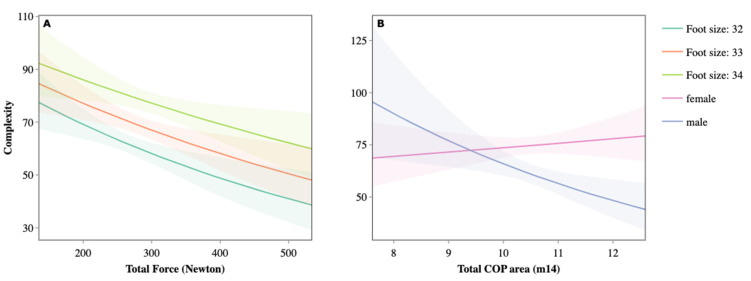
Variation in the complexity score as a function of total strength (**A**), COP, and sex (**B**).

Finally, the COP area is an element that affects the complexity score, with an unequal relationship between sexes. In boys, the greater the area, the lower the balance score, while in girls, the complexity was independent of the variation in COP (Figure 7).

### 3.5. Design of Scoring Scales

After performing the total motion study in the SLB test, the maximum value of motion recorded was 347.53. With this, it is decided to apply *cte* = 350 to invert the scale and obtain the raw score. In the case of the TBT, the maximum value was 288.80 and its value *cte* = 300. Finally, in the YBT, a maximum value of 84 was obtained, with a value *cte* = 80.

$$Raw\;score\;SLB=-\left(mvt\;exercise\;a+mvt\;exercise\;b+mvt\;exercise\;c+mvt\;exercise\;d\right)+350$$

$$Raw\;score\;TBT=-\left(mvt\;exercise\;a+mvt\;exercise\;b+mvt\;exercise\;c+mvt\;exercise\;d\right)+300$$

$$Raw\;score\;YTB=log\left(YBT\;a+YBT\;b+YBT\;c+YBT\;d+YBT\;e+yby\;f-80\right)$$

The linear model to determine the variation in the raw score from sex, age, and foot size resulted in the following formulas, from which the correction factor, FC, was obtained:

$$SLB\;FC=1053.32+6.03\cdot \left(Sex==male\right)-53.41\cdot Age-12.46\cdot Foot\;size$$

$$TBT\;FC=1009.87+10.96\cdot \left(Sex==male\right)-54.58\cdot Age-11.90\cdot Foot\;size$$

$$YBT\;FC=1.61+0.11\cdot \left(Sex==male\right)+0.05\cdot Age-0.04\cdot Foot\;size$$

**Figure 8:**
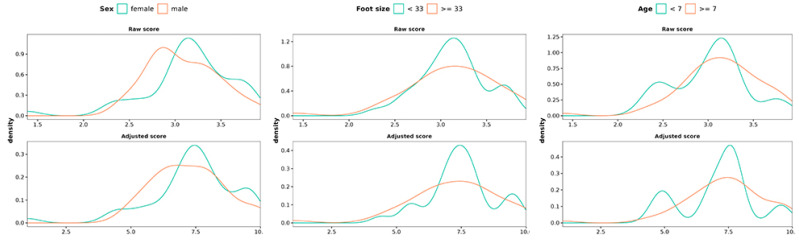
Raw score for the total amount of movement, together with adjusted and rescaled values for sex (**left**), foot size (**center**), and age (**right**).

By applying the correction factor to the raw scores obtained for the population studied, it was determined that the range of these adjusted scores is, in the case of the SLB, from −72 to 230; for the TBT, from −54 to 206; and finally, for the YBT, from −175 to 526. Figure 8 shows the raw score obtained from the total amount of movement and adjusted and rescaled values on YBT.

## 4. Discussion

The objective of this study was to design a free application for the assessment of static and dynamic balance using a force platform for children between 6 and 9 years of age. The use of mobile applications to monitor fitness indicators has become widespread, providing immediate information on different variables to users with few instructions, even in gamified form [59], for various purposes [60].

As indicated by Kabir et al. [61], the current reliability of these applications is very low, and the accuracy of the data received is essential for their correct interpretation and subsequent decision-making [62]. It is important that they are intuitive, simple, and allow quick understanding [63] based on validated instruments to favor the programming of conditioning work [64].

Balance is fundamental in the perceptual–motor phase, since, in addition to the work on the vestibular system, it integrates determining social functions in preadolescence. Moreover, it is part of all general motor patterns of both basic and more sophisticated techniques, helping the correct location in space in a prominent manner when visual tasks are added [65]. Therefore, it is important to identify whether there is an adequate level of development statically or dynamically, and to observe possible anomalies or dysfunctions in relation to the age of measurement [66], and in this case, the difference in performance competence can be a useful indicator.

A greater complexity was found in the tests performed with closed eyes (SLB and TBT), with a greater height and standing height, possibly due to a greater COM height (Table 3).

In the case of YBT, beyond the complexity, joint mobility is relevant. A greater posteromedial and posterolateral mobility are linked to a greater strength in the lower extremities in 7-year-old children [67]. In this study, it was added to this information that the subject profile with a greater leg length facilitates a better balance in the anterior direction and less in the posterolateral direction.

In the case of the COP, in girls, a greater variation means a better score in the balance variable, as opposed to boys, with less fluctuation due to the difference in the base of support (foot size). Furthermore, it can be affirmed that, in the case of girls, with a lower application of force, they increase the global complexity by having a greater variability in the COP, unlike the boys, whose greater total force decreases this variable for the achievement of balance. Therefore, it can be deduced that a greater movement in girls is the reason for a higher score, unlike in boys (Figure 7).

In addition to evaluating the level of static or dynamic balance and the degree of complexity, these tests are useful for identifying ankle instability by the level of applied force and the detection of a lower participation in physical activities, with possible negative consequences for their health [68]; therefore, they are an excellent reference for Physical Education teachers in the selection of tasks to be developed. The greater or lesser total strength identified in relation to the variation in the COP opens a line of preventive work [69] that would allow correcting the variations that occur, with a greater assistance in the case of girls.

In this line, the observational tool Kinovea, used in similar work with children [70], captured the interrelation between interaxial forces and postural control, allowing for the establishment of a scale of easy interpretation after a sequence of four phases: (1) calculation of movement at reference points, (2) calculation of total movement and raw score, (3) score adjustment attending to demographics, and (4) rescaling to a scale with a score from 1 (greatest imbalance) to 10 (greatest balance), with which to differentiate the level of development by age and sex [71] (unpublished data). These procedures are in line with other studies that have used conventional cameras for kinematic calculations of balance [72] and are similar to those proposed in the Berg scale [73], except that these and other similar designs were intended for special populations without differentiating the specific characteristics of children [59].

This study has several limitations. It should be noted that the age range was small, and the measurement of such young people can be complex. The application requires bridging software to be used to change position (Kinovea, MyLab, or the mobile device’s measurement level widget). Finally, it is necessary to apply it in school courses to determine its accessibility and applicability in the educational environment, which would be facilitated by the conversion of the responsive web application into a native mobile app for the two most used operating systems. In any case, it allows the measurement of several tests (dynamic and static) to collect periodic monitoring by keeping data from several dates and to compare, by age, the perceptual–motor evolution of the children.

## 5. Conclusions

After this investigation, it can be affirmed that static balance is more complex with a greater age, greater standing height, and keeping the eyes closed without significant changes. For dynamic balance, the highest score was associated with anterior mobility and leg length.

With respect to the COP, its variability is greater in girls than in boys, who present less complexity when maintaining balance due to a greater total force at the time of the test.

Finally, the objectivity of the data collected on the force platform, together with the observation with Kinovea, allows the elaboration of an easily interpretable scoring scale, whose environment is an open-access web application (IMEP) to objectify static and dynamic balance tests.

## Figures and Tables

**Table 1:** Characteristics of the students participating in the study (mean ± standard deviation).

		Global	Female	Male
N		75	44	31
Age		7.27 ± 0.74	7.23 ± 0.71	7.32 ± 0.79
Weight (kg)		27.25 ± 7.37	25.91 ± 5.93	29.16 ± 8.79
High (m)		1.28 ± 0.06	1.27 ± 0.05	1.29 ± 0.08
BMI		16.59 ± 3.64	16.1 ± 3.2	17.29 ± 4.14
Dominant foot	Right	68 (90.67%)	42 (95.45%)	26 (83.87%)
Non dominant foot	Left	6 (8%)	2 (4.55%)	4 (12.9%)
Foot Size		32.95 ± 2.14	32.48 ± 1.66	33.63 ± 2.56
Leg length		67.45 ± 4.62	67.28 ± 4.5	67.69 ± 4.85

Note: BMI = Body mass index. SD = Standard deviation.

**Table 2:** Evaluation of the balance test complexity.

Variable	χ2	Df	*p*-Valor
(Intercept)	5.402	1	0.02 *
Standing foot	0.578	1	0.447
Age	78.617	1	7.5 × 10^−19^ ***
Type of test	44.931	2	1.8 × 10^−10^ ***
Foot size	12.605	1	0.00038 ***
Sex	0.479	1	0.489
Open eyes	4.492	1	0.034 *
Type of test: Foot size	29.247	2	4.5 × 10^−7^ ***
Type of test: Sex	4.6	2	0.1
Foot Size: Open eyes	2.984	1	0.084

Note: * = *p* < 0.05. *** = *p* < 0.001.

**Table 3:** Effect of the type of movement performed on YBT.

YBT	Emmean (95%)	Contrast	Estimate	t Ratio	*p* Value
Anterior	18.57 ± 0.23 [18.11–19.03]	Anterior–Posterolateral	2.27 ± 0.22	10.45	0.0 ***
Posterolateral	16.3 ± 0.23 [15.84–16.76]	Anterior–Posteromedial	1.09 ± 0.22	5.04	2.2 × 10^−6^ ***
Posteromedial	17.47 ± 0.23 [17.01–17.93]	Posterolateral–Posteromedial	−1.18 ± 0.22	−5.41	3.4 × 10^−7^ ***

Note: *** = *p* < 0.001.

**Table 4:** Balance ratio according to foot size and sex.

Sexo	Test	Emmean	Contrast	Estimate	t Ratio	*p* Value
Female	SLB	158.89 ± 9.55 [141.21–178.78]	SLB/TBT	0.78 ± 0.07	−2.82	0.014 *
Female	TBT	202.54 ± 13.53 [177.65–230.92]	SLB/YTest	1.07 ± 0.1	0.77	0.724
Female	YTest	147.93 ± 10.09 [129.4–169.11]	TBT/YTest	1.37 ± 0.14	3.06	0.006 **
Male	SLB	118.96 ± 8.65 [103.13–137.21]	SLB/TBT	0.91 ± 0.09	−0.87	0.662
Male	TBT	130.04 ± 10 [111.82–151.21]	SLB/YTest	0.8 ± 0.09	−1.99	0.116
Male	YTest	148.68 ± 12.21 [126.56–174.68]	TBT/YTest	0.87 ± 0.1	−1.13	0.497

Note: * = *p* < 0.05. ** = *p* < 0.01.

**Table 5:** Complexity by gender and type of test.

Sex	Test	Emmean	Contrast	Estimate	t Ratio	*p* Value
Female	SLB	63.56 ± 3.07 [57.82–69.88]	SLB/TBT	1.07 ± 0.07	1.01	0.570
Female	TBT	59.28 ± 3.28 [53.18–66.09]	SLB/YTest	0.58 ± 0.04	−7.47	8.5 × 10^−13^ ***
Female	YTest	109.76 ± 5.65 [99.21–121.43]	TBT/YTest	0.54 ± 0.05	−7.36	1.4 × 10^−12^ ***
Male	SLB	55.71 ± 3.43 [49.37–62.87]	SLB/TBT	1.36 ± 0.11	3.74	5.6 × 10^−4^ ***
Male	TBT	40.96 ± 2.76 [35.89–46.75]	SLB/YTest	0.54 ± 0.05	−7.07	9.2 × 10^−12^ ***
Male	YTest	102.41 ± 6.22 [90.9–115.38]	TBT/YTest	0.4 ± 0.04	−9.69	3.2 × 10^−13^ ***

Note: *** = *p* < 0.001.

**Table 6:** Complexity according to applied force and foot size.

Foot Size	TF	Emmean	Contrast	Estimate	t Ratio	*p* Value
32	229.1	65.75 ± 2.13 [61.69–70.07]	TF 229.12/TF 265.59	1.07 ± 0.02	3.41	0.002 **
32	265.6	61.69 ± 1.73 [58.4–65.18]	TF 229.12/TF 318.31	1.17 ± 0.05	3.41	0.002 **
32	318.3	56.27 ± 2.27 [51.99–60.91]	TF 265.59/TF 318.31	1.10 ± 0.03	3.41	0.002 **
33	229.1	74.03 ± 2.47 [69.34–79.03]	TF 229.12/TF 265.59	1.05 ± 0.02	3.16	0.005 **
33	265.6	70.30 ± 1.79 [66.88–73.91]	TF 229.12/TF 318.31	1.13 ± 0.05	3.16	0.005 **
33	318.3	65.25 ± 1.99 [61.46–69.27]	TF 265.59/TF 318.31	1.08 ± 0.03	3.16	0.005 **
34	229.1	83.35 ± 3.21 [77.29–89.89]	TF 229.12/TF 265.59	1.04 ± 0.02	2.55	0.030 *
34	265.6	80.12 ± 2.30 [75.72–84.77]	TF 229.12/TF 318.31	1.10 ± 0.04	2.55	0.030 *
34	318.3	75.66 ± 2.05 [71.75–79.79]	TF 265.59/TF 318.31	1.06 ± 0.02	2.55	0.030 *

Note: TF = Total force. * = *p* < 0.05. ** = *p* < 0.01.

## Data Availability

The datasets used and analyzed during the current study are available from the corresponding author upon reasonable request due to privacy and ethical restrictions.

## Abbreviations

COM = Centre of mass; COP = Centre of pressure; SLB = Single leg balance test; TBT = Tandem balance test; YBT = Y balance test; SEBT = Star excursion balance test; TF = Total force; IMEP = Instrumento para la Medición del Equilibrio en Primaria.
